# The Treatment of Typhoid Fever

**Published:** 1892-09

**Authors:** 


*The Treatment of Typhoid Fever.* By JAMES BARR, M.D., Physician to the Northern Hospital, Liverpool. Pp. x., 212. London: H. K. LEWIS. 1892.

If we are somewhat late in noticing Dr. Barr's book, it is not because the work is otherwise than most interesting and suggestive. The special feature of his treatment consists in keeping the patient continuously immersed in a tank of water at a temperature of about 90°, or a little higher if the temperature of the patient is approaching the normal. The excellence of the results of this method of treatment upon the course of the febrile process itself, and particularly upon the nervous condition of the patients, is described with admirable detail. Medicinal antipyretics Dr. Barr does not use, considering them to be agents more for evil than for good; alcohol, also, he employs with exceeding rarity. Intestinal antiseptics, on the other hand, he places confidence in, and notes Dr. J. Michell Clarke's approbation of hydronaphthol for this purpose. The diet which he permits is abundant, possibly some might think over-abundant: "I place no limit on the quantity of liquid nourishment, so long as it is retained in the stomach and properly digested; but for an adult I usually find that about four pints of milk, eight to sixteen ounces of bread, and two ounces of butter, are appropriated daily. The bread should be boiled with the milk, or it may be peptonised and the butter added. In the tank the appetite soon becomes very good, and these quantities do not suffice!" A careful consideration of the method will undoubtedly reveal that it is not altogether without drawbacks, and must present considerable difficulty in being carried out in private practice; still the results are strikingly successful, and as Dr. Barr well remarks, "where human life is concerned no obstacle should impede our path." The book is written in clear, concise language, free from exaggeration or vagueness, and is eminently deserving of attentive perusal.

